# A systematic mapping review of Randomized Controlled Trials (RCTs) in care homes

**DOI:** 10.1186/1471-2318-12-31

**Published:** 2012-06-25

**Authors:** Adam L Gordon, Phillipa A Logan, Rob G Jones, Calum Forrester-Paton, Jonathan P Mamo, John RF Gladman

**Affiliations:** 1 Division of Rehabilitation and Ageing, University of Nottingham, Nottingham, UK; 2 Department of Psychiatry, University of Nottingham, Nottingham, UK; 3 Department of Health Care of Older People, Nottingham University Hospitals NHS Trust, Nottingham, UK; 4 Department of Medicine, Peterborough City Hospital, Peterborough, UK

## Abstract

**Background:**

A thorough understanding of the literature generated from research in care homes is required to support evidence-based commissioning and delivery of healthcare. So far this research has not been compiled or described. We set out to describe the extent of the evidence base derived from randomized controlled trials conducted in care homes.

**Methods:**

A systematic mapping review was conducted of the randomized controlled trials (RCTs) conducted in care homes. Medline was searched for “Nursing Home”, “Residential Facilities” and “Homes for the Aged”; CINAHL for “nursing homes”, “residential facilities” and “skilled nursing facilities”; AMED for “Nursing homes”, “Long term care”, “Residential facilities” and “Randomized controlled trial”; and BNI for “Nursing Homes”, “Residential Care” and “Long-term care”. Articles were classified against a keywording strategy describing: year and country of publication; randomization, stratification and blinding methodology; target of intervention; intervention and control treatments; number of subjects and/or clusters; outcome measures; and results.

**Results:**

3226 abstracts were identified and 291 articles reviewed in full. Most were recent (median age 6 years) and from the United States. A wide range of targets and interventions were identified. Studies were mostly functional (44 behaviour, 20 prescribing and 20 malnutrition studies) rather than disease-based. Over a quarter focussed on mental health.

**Conclusions:**

This study is the first to collate data from all RCTs conducted in care homes and represents an important resource for those providing and commissioning healthcare for this sector. The evidence-base is rapidly developing. Several areas - influenza, falls, mobility, fractures, osteoporosis – are appropriate for systematic review. For other topics, researchers need to focus on outcome measures that can be compared and collated.

## Background

Care homes provide accommodation, together with nursing or personal care, for persons who are or have been ill, who have or have had a mental disorder, who are disabled or infirm, or are or have been dependent on alcohol or drugs [1]. In the UK, 91 % of residents are over 70 years of age, 76 % require assistance with mobility or are immobile and 78 % have at least one form of mental impairment [2].

In some countries, such as the Netherlands and USA, health care professionals are based within, or employed by, care homes [3-5]. In others, such as the UK or Ireland, the health care provision for care homes is provided by generic primary care services. The provision of health care to this sector is a matter of concern, debate and innovation [4-8].

Quest for Quality, a document by the British Geriatrics Society [6], stated that health care for care homes in the UK was characterised by “unmet need, unacceptable variation and often poor quality of care”. To improve matters, the report recommended the development of a more structured and evidence-based approach to commissioning. To inform this process, we set out to describe the extent of the evidence-base for the effectiveness of interventions specific to care home residents. We chose a systematic mapping review because these are specifically designed to describe the extent of research in a field [7,8]. We chose to review only randomised controlled trials (RCT) so that the findings would represent the highest tier of medical evidence for therapeutic interventions [9].

## Methods

Medline (1950-Jun 2009) was searched for “Nursing Home”, “Residential Facilities” and “Homes for the Aged”, combined using the “OR” command. Results were limited for English language and RCTs. CINAHL with full text (1978-June 2009) was searched for “nursing homes”, “residential facilities”, “skilled nursing facilities”, with results limited to RCTs. The Allied and Complementary Medicine Database (AMED) (1985-June 2009) was searched for “Nursing homes”, “Long term care” and “Residential facilities” combined using the “OR” command and “Randomized controlled trial” using the “AND” command. The British Nursing Index and Archive (BNI) (1985-June 2009) was searched for “Nursing Homes”, “Residential Care” and “Long-term care”. Abstracts were reviewed by a single researcher and articles included if they described interventions evaluated by RCT in residential, nursing or care homes.

A keywording strategy [8] was developed by three researchers using an iterative approach and a random sample of 20 articles, which were reviewed repetitively with key descriptors recorded. The researchers met after each iteration and the process concluded when two subsequent reviews identified no new descriptors. The resulting framework described: year of publication, country of publication, individual or cluster randomization, stratified or non-stratified randomization, method of stratification, blinding strategy (patient/investigators/both/neither), target of intervention, intervention treatment, control treatment, number of subjects (total/intervention/control), number of clusters (total/intervention/control), outcome measures and results. The remaining articles were then divided amongst six reviewers who classified them according to the keywording strategy. As a final measure, all articles were reviewed by the lead researcher with disagreements resolved by consensus.

## Results

**Figure 1:**
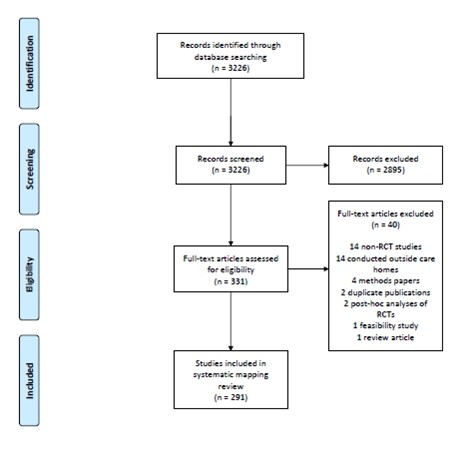
PRISMA flow diagram.

The abstracts of the 3226 unique citations identified from the search were examined, 331 of which described RCTs in care homes. Forty of these were excluded at full review leaving 291 articles in this review. A PRISMA flow diagram is shown in Figure 1.

The majority of studies (145) were conducted in the USA, followed by the UK (24) and the Netherlands (23): 163 articles came from the Americas, 87 articles from Europe, 23 from Asia and 16 from Australasia.

**Figure 2:**
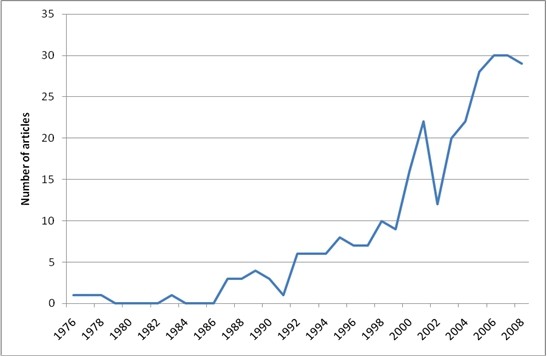
Number of articles published by year.

Figure 2, showing the publication rate by year, demonstrates a steady increase in the number of publications over the last two decades. The median age of the publications was 6 years.

Key methodological attributes of the studies are outlined in Table 1.

**Table 1:** Methodological attributes of studies

	**Blinding**			
	**Double blind**	**Participant blinded only**	**Outcome assessor blinded only**	**Unblinded**
Cluster	6	0	18	44
Cluster Crossover	1	0	5	3
Individual Patient	70	5	42	74
Individual Patient Crossover	8	2	7	7

36 primary targets for interventions were identified, as summarised in Table 2.

**Table 2:** Targets of interventions

**Target and type of interventions**	**Number of studies**	**Number of participants**
Behaviour	44	4482
Pharmacological [10-28]	19	2521
Occupational therapy, aids and appliances [29-35]	7	471
Physical therapy [36-39]	4	311
Staff education [40-43]	4	301
Psychological or behavioural Therapy [44-47]	4	286
Case management [48-51]	4	449
Nursing, not classified elsewhere [52]	1	73
Aromatherapy [53]	1	70
Prescribing	20	17679
Pharmacological [54-67]	14	13241
Staff education [68-73]	6	4438
Malnutrition	20	1859
Nutritional [74-89]	16	1382
Pharmacological [90-93]	4	477
Influenza	19	7720
Vaccination [94-106]	13	5314
Pharmacological [107-112]	6	2406
Quality of Life	18	2557
Psychological or behavioural therapy [113-116]	4	221
Occupational therapy, aids and appliances [117-119]	3	208
Physical therapy [120-122]	3	299
Staff and family education [123-125]	3	1419
Care home administration [126,127]	2	81
Case management [128]	1	106
Nutritional [129]	1	178
Nursing, not classified elsewhere [130]	1	45
Depression	17	888
Physical therapy [131-139]	9	518
Pharmacological [140-143]	4	176
Psychological or behavioural therapy [144,145]	2	87
Case management [146]	1	85
Occupational therapy, aids and appliances [147]	1	22
Mobility	13	1435
Physical therapy [148-157]	10	615
Occupational therapy, aids and appliances [158-160]	3	820
Oral Health	13	1279
Dental and oral health interventions [161-172]	12	1255
Pharmacological [173]	1	24
Falls	12	4922
Occupational therapy, aids and appliances [174-179]	6	2677
Pharmacological [180,181]	2	749
Physical therapy [182,183]	2	207
Care home administration [184]	1	910
Staff education [185]	1	379
Quality of Care	12	11947
Care home administration [186-193]	8	10417
Staff education [194-196]	3	1338
Case management [197]	1	192
Urinary incontinence	12	896
Occupational therapy, aids and appliances [198-203]	6	410
Pharmacological [204-207]	4	216
Physical therapy [208]	1	190
Nursing, not classified elsewhere [209]	1	80
Cognitive performance	7	536
Physical therapy [210-212]	3	125
Pharmacological [213,214]	2	371
Psychological or behavioural therapy [215,216]	2	40
Sleep	8	488
Physical therapy [217-223]	7	474
Pharmacological [224]	1	14
Fractures	8	10221
Occupational therapy, aids & appliances [225-232]	8	10221
Immunity	8	1304
Pharmacological [233-237]	6	890
Nutrititional [238]	1	257
Physical therapy [239]	1	157
Decubitus Ulcers	7	658
Occupational therapy, aids and appliances [240,241]	2	116
Pharmacological [242,243]	2	145
Physical therapy [244,245]	2	162
Nursing, not classified elsewhere [246]	1	235
Osteoporosis	7	14096
Pharmacological [247-251]	5	7853
Care home administration [252]	1	5637
Staff education [253]	1	606
Pain	7	1038
Case management [254,255]	2	232
Physical therapy [256,257]	2	73
Psychological or behavioural therapy [258]	1	21
Pharmacological [259]	1	39
Staff education [260]	1	673
Physical Function	6	726
Occupational therapy, aids and appliances [261-264]	4	368
Care home administration [265]	1	164
Physical therapy [266]	1	194
Constipation	5	494
Nutritional [267-270]	4	337
Physical therapy [271]	1	157
Respiratory infection	5	1724
Pharmacological [272-275]	4	1063
Care home administration [276]	1	661
Physical Restraint Use	3	758
Staff education [277-279]	3	758
Skin Health	3	205
Nursing, not classified elsewhere [280,281]	2	61
Physical therapy [282]	1	144
Vitamin D deficiency	3	217
Pharmacological [283,284]	2	172
Physical therapy [285]	1	45
General health	3	585
Care home administration [286]	1	464
Case management [287]	1	69
Pharmacological [288]	1	52
Swallowing	2	78
Pharmacological [289]	1	63
Physical therapy [290]	1	15
Compliance with OT [291]	1	30
COPD [292]	1	89
Cough Reflex Sensitivity [293]	1	59
Dehydration [294]	1	63
Dementia [295]	1	426
Faecal Incontinence [296]	1	178
Hypertension [297]	1	30
Interpersonal skills [298]	1	27
Microbial colonisation [299]	1	283
UTI [300]	1	50

For interventions targeting resident behaviour, most pharmacological studies evaluated risperidone [22-26] or olanzapine [18,19]. Two studies evaluated interventions aimed at withdrawal of antipsychotic medications [27,28]. Studies listed under occupational therapy, aids and appliances targeting behaviour were heterogeneous and included Activities of Daily Living (ADL)-targeted interventions [29,30], re-orientation [31], pet therapy [32] and music therapy [33-35]. Physical therapy studies evaluated either light therapy [37-39] or exercise therapy [36]. Staff education interventions focussed either around communication [41-43] or goal-setting [40].

Seven studies targeting prescribing of medications looked at incorporating pharmacist review, with or without physician involvement, into clinical pathways on or after admission to care home [55-57,61-63,65]. Other, more targeted interventions considered protocols for the withdrawal of hypnotics [54], neuroleptics [59,66], anti-depressants [58] and anti-Parkinsonian [67] drugs. Two studies evaluated the effects of specific antibiotic protocols on prescribing [60,64]. Six studies evaluated the impact of staff education on prescribing, three considered teaching on psychopharmacology [69,70,73], one on generic prescribing issues [72], one on antibiotics [68] and one on pain management [71].

The majority of studies targeting nutrition evaluated nutritional supplementation using vitamin, mineral, and/or protein-energy supplementation [78,79,81-89,93]. Three studies, all from the same authors, evaluated the effect of megestrol acetate on nutritional biomarkers [90-92]. Other studies evaluated the effect of flavour enhancers [77], dietary restriction [80], family-style dining arrangements [75,129] and the provision of feeding assistance [76].

Studies targeting influenza in residents predominantly compared doses or types of vaccine [94-96,101,102,104-106]. Three studies compared influenza vaccination with placebo [97,99,100] and two with usual care [98,103]. Six evaluated neuraminidase inhibitors [107-112].

Studies targeting quality of life were heterogeneous. Under this heading, studies evaluating psychological or behavioural interventions evaluated group or individual therapies to build self-esteem [113,114] or reminiscence therapy [115,116]. Occupational therapy-oriented studies to improve quality of life evaluated spectacle correction of eyesight, engagement in teaching and pets [117-119]. Physical therapy studies to improve quality of life evaluated tai chi, back rubs and functional incidental training [120-122]. Staff training interventions to improve quality of life evaluated teaching about end-of-life care, dementia management and conflict resolution [123-125].

Studies targeting depression evaluated exercise therapy [132-134,136,137], light therapy [131] and yoga [138]. There were four studies of antidepressants [140-143], one of reminiscence [144] and one of self worth therapy [145].

Interventions targeting mobility were much more homogenous. Ten physical therapy studies all evaluated forms of exercise therapy [148-157] while two of the three occupational therapy interventions evaluated multi-faceted mobility interventions [158,159]. The final study evaluated visual feedback balance training [160].

Oral health studies predominantly evaluated the role of mouthwashes, toothpastes and other oral preparations in dental hygiene [161,162,165,166,169] but also evaluated toothbrushing technologies and techniques [164,170,171], oral healthcare education [168,172], restorative dentistry [163] and denture care [167]. One study evaluated subantimicrobial doses of an antibiotic as a treatment for chronic periodontitis [173].

The majority of studies that evaluated falls prevention interventions focussed on multifaceted programmes [174-179]. Two studies evaluated the role of vitamin D supplementation [180,181] and two evaluated exercise therapy [182,183]. One evaluated the role of care home governance in falls documentation [184] and one evaluated the impact of staff education on falls rates [185].

Studies targeting quality of care focussed predominantly on care home administration: four examined quality assurance programmes [189-192], two the implementation of advanced care planning or advanced directives [186,187], one communication with families [188] and one resident relocation [193]. The remaining studies evaluated the impact of staff education on bathing [194], communication [195] and death and dying [196].

Of the less frequently studied areas, large cohorts were seen in studies targeting fractures and osteoporosis. These areas were also notable for the similarity of the interventions studied and outcome measures recorded: all eight fracture studies evaluated hip protectors [225-232] and four [248-251] out of seven osteoporosis studies evaluated calcium and vitamin D therapy, with or without bisphosphonates, whilst a further study evaluated alendronate as a standalone therapy [247].

Ten studies evaluated case management in the care home setting. The effect of psychiatric case management was evaluated in six studies, two on behavioural disturbance [49,51], two on depression [51,146] and two on quality of life [128,197]. Four studies evaluated broader assessment and management taking account of somatic, psychological and environmental issues, which might be termed comprehensive geriatric assessment [48,254,255,287]. One study evaluated protocol-driven disease management for COPD by care home nurses [292].

## Conclusions

These findings provide a unique resource for those providing health care to the care home sector, collating for the first time the range of interventions and outcomes that have been tested using RCTs specifically in care homes. There have been a considerable number of RCTs of interventions specifically in care homes (292 between 1974 and 2009). These studies are relatively recent, since half of them were published in the last 6 years. They evaluated a diverse range of interventions such as light therapy, staff training and oral hygiene and an equally diverse range of targets such as reducing falls, increasing weight or improving mood. The studies were mostly functional (44 behaviour studies, 20 prescribing studies, 20 malnutrition studies) rather than disease based (1 study each for UTI and COPD). Over a quarter of papers focussed on mental health or behaviour. Despite our use of systematic searching, we found no previously published studies that had collated this literature.

A limitation of this review is the geographical distribution of papers, with the majority of studies arising from the USA and relatively few from non-English speaking nations, thus reducing the generalisability of the findings worldwide. Our decision to select only RCTs means that we have not collated the entire evidence base for interventions in care homes and are likely to have missed several important studies as a consequence. Such studies would include those focussing on policy, facility organizational characteristics and the ways in which care homes interact with other care services, including the health service – these have been recognised to be important drivers of care quality but have been studied by means other than RCT [6].

It is possible that staff trained in the care home setting – as some doctors are in the Netherlands or USA – are aware of many of these studies but it is less likely that those who provide health care to care homes as part of generic primary care will be familiar with them. The recent growth in the number of papers means that even health care professionals with an interest in the area will be out of date if they have not recently consulted the literature. The wide-ranging nature of the studies identified will have relevance to commissioners, and health care professions including general practitioners, geriatricians, psychiatrists, nurses, occupational, physio- and speech and language therapists, dieticians, dentists, pharmacists and social workers – as well as care home staff. From a research perspective, there were few targets where multiple studies evaluated similar interventions against a common outcome measure. The obvious exceptions were influenza, falls, mobility, fractures and osteoporosis and these areas are suitable for systematic reviewing. There was great diversity of interventions and targets, such that there were few papers using the same intervention and the same target. Thus, despite the large numbers of papers found, much work remains to be done to develop a robust evidence-base for each intervention and each target. Although a detailed methodological critique is beyond the scope of a systematic mapping review, the fact that the majority of studies were unblinded and did not employ cluster randomization means that many will have been methodologically flawed.

In summary, these findings represent an important resource for commissioners, clinicians and researchers involved with care homes. At the very least, the diversity of interventions and outcomes illustrated should challenge nihilism towards this health care sector and encourage further innovation.

## Competing interests

The authors have no competing interests to declare.

## Authors' contributions

ALG was lead researcher and also lead author. JRFG and PAL contributed to the development of the keywording strategy. All authors participated in the literature review and contributed to drafting the final manuscript. All authors read and approved the final manuscript.

## Pre-publication history

The pre-publication history for this paper can be accessed here:

http://www.biomedcentral.com/1471-2318/12/31/prepub
